# Potential effects of using non-combustible tobacco and nicotine products during pregnancy: a systematic review

**DOI:** 10.1186/s12954-020-00359-2

**Published:** 2020-03-02

**Authors:** M. Glover, Carl V. Phillips

**Affiliations:** 1 Centre of Research Excellence: Sovereignty & Smoking, 8 Toroa Street, Torbay, Auckland, 0632 New Zealand; 2 New Hampshire, USA

**Keywords:** Tobacco, Nicotine, Pregnancy, Snus, Snuff, Vaping, Chewing tobacco, Nicotine replacement therapy, Review

## Abstract

**Background:**

The range of risk reduced alternatives to smoking tobacco is increasing and so is use among pregnant women. The substantial harms of smoking during pregnancy are well established and there is reason to believe that nicotine alone is somewhat harmful. Differences in the exposure chemistry strongly suggest that the effects of using smoke-free nicotine products (including pharmaceutical nicotine products, smokeless tobacco, and electronic cigarettes containing nicotine) fall somewhere in the range between zero risk to the risk from smoking. How much lower risk these consumption choices are in terms of pregnancy outcomes, however, remains uncertain.

**Methods:**

We reviewed the literature on smoke-free nicotine and tobacco product exposure and birth-outcome endpoints. Studies were included if they compared outcomes to either no nicotine use or smoking. We searched Google Scholar using broad search terms and additional articles were snowballed from citations. We report what could be learned from each study, given its methods.

**Results:**

Of the 21 studies reviewed, 12 reported on the use of nicotine replacement therapies, 7 on Swedish snus, 1 on Alaskan iq’mik, and 1 on e-cigarettes. The range of results tends to support the prediction that smoke-free product use during pregnancy probably increases the risk of some negative birth outcomes, but that any effect is less than that from smoking. However, the limitations of epidemiology are such that no more-precise a conclusion is possible.

**Discussion:**

The available epidemiology does not change our prior beliefs, based on other evidence and knowledge, that the risks from smoke-free nicotine and tobacco are lower than those for smoking, though it suggests they are non-zero. However, it also demonstrates that the epidemiology is unlikely to provide precise quantitative estimates. This is not just a matter of lack of studies; given the inherent limitation of these studies, doubling or tripling the corpus of available studies would add little precision. For the foreseeable future, decisions about using these products will need to be made based on rough estimates, based on a variety of forms of evidence, and qualitative comparisons.

## Background

The harms of smoking during pregnancy are well established [1, 2]. The clearest effect is on birth weight (though it is widely believed this reduction is not as harmful as naïve associations would predict—i.e., smoking causing lower birth weight does not cause risks nearly as great as those for the average baby with that lower birth weight) [3]. But there also appear to be measurable effects on preterm delivery, malformations, childhood respiratory disease, and increased risk of childhood cancers. Pregnant women are widely advised to avoid all lifestyle drugs, including nicotine, caffeine, and alcohol. But some women who smoke will not achieve abstinence when pregnant. One review estimated that approximately 8% of pregnant women in Europe and 6% in the Americas continued smoking [2].

For smokers who do not choose to abstain from nicotine, their own health can be improved—by approximately as much as by becoming abstinent—by switching to smoke-free nicotine products. There is every reason to believe such substitution would also be beneficial for the fetus. Eliminating the carbon monoxide and particulate matter that enters the bloodstream from smoke alone seems certain to be better for the fetus. But there are also reasons to believe that nicotine alone has effects on pregnancy. For example, some studies have found that nicotine alone causes developmental abnormalities in animals. In vitro studies have suggested teratogenic effects of nicotine exposure. The vasoconstrictive effect of nicotine could negatively affect fetal development.

Low-risk smoke-free alternatives include pharmaceutical smoking cessation products (usually called “nicotine replacement therapy” (NRT))—patches, gums, lozenges, and other nicotine delivery systems. These are commonly used for ongoing nicotine delivery. Low-risk alternatives also include smokeless tobacco—chewing tobacco, snuff and snus (the Swedish word for oral snuff). Snus historically was used almost exclusively by men in Scandinavia but has become increasingly popular with women there. Smokeless tobacco use in North America is still almost exclusively male. These oral or chewing products used in Scandinavia and North America should not be confused with various products that are widely used elsewhere, such as in India. Some of these pose unknown and apparently higher risk because many are not actually even tobacco, or the tobacco content is a minor ingredient in what is primarily areca (also called betel) nut, slaked lime, ash or other plant matter or spices. These products are seldom, if ever, recommended as substitutes for smoking. Over the last decade, vaping (electronic cigarette use) has emerged as the most popular smoke-free nicotine source in many populations. Tobacco heating products (also called heat-not-burn) have become popular in some populations. There is legitimate uncertainty about whether they are quite as low-risk as the other products. There are also a variety of other less popular smoke-free and presumably low-risk products, though none appear in our review.

It is easy to conclude that the pregnancy risks from using smoke-free products must be less than those from smoking, probably far less. Analytic chemistry shows that the exposures to potentially harmful chemicals are almost a proper subset of those from smoking (i.e., almost every exposure from using smoke-free nicotine products is present in smoking, and the exceptions are believed to be benign). Most of the potentially harmful exposures from smoking are absent or dramatically reduced in the smoke-free products. The available toxicology confirms the predictions we would make from this. We know that inhaling smoke—whatever is burning—is harmful in itself. We also know from the epidemiology of Scandinavian and Western smokeless tobacco that any health risks to the user herself are below the threshold of what epidemiology can detect, and this seems likely to translate into lower risk for the fetus [4, 5]. Still, there is ample toxicological evidence that nicotine itself is not benign for the developing fetus, and other aspects of the smoke-free product exposure may have negative effects on the fetus even if they pose no measurable health risk for the woman herself.

This brings up the question of whether it is possible to quantify how much less risky using a smoke-free alternative is, in terms of pregnancy outcomes, compared to smoking. A 2008 review, by the European Scientific Committee on Emerging and Newly Identified Health Risks [6], concluded there was inadequate evidence to quantify the pregnancy risks from snus and other low-risk products. Another review of epidemiological evidence of snus use found very limited scientific evidence of harms of using snus during pregnancy [7]. Three other reviews reported on potentially harmful effects of NRT use during pregnancy [8–10]. Several other reviews are difficult to interpret because it is impossible to assess their methods [11–15]. All of these reviews present some quantitative results, but the overall picture was one of inadequate information.

## Methods

The goal of the present analysis was to conduct a review of the accessible epidemiologic evidence about the pregnancy outcome effects of all smoke-free nicotine and tobacco products to assess (a) what are our best current available quantitative estimates and (b) what is the potential (or lack thereof) for such research to provide precise quantitative estimates. Specified outcome measures appear as the subsections in the results.

### Study design

A literature review was conducted between January 2019 and April 2019. Studies were included in this review if they purported to compare birth outcomes for smoke-free product exposure (or an attempt to encourage such exposure) to either no product use or to smoking. The PRISMA protocol [16] was used to guide the design of our review. The PRISMA protocol assumes that systematic reviews will focus on how to optimally interpret the reviewed quantitative results. Whilst that was our plan, the quality of the literature prohibited this. During the analysis, we discovered that most of the results in the literature could not be used to produce the required PRISMA level of detail or valid PRISMA level of standard interpretations.

### Search strategy

We searched Google Scholar, the most comprehensive collection of scholarly papers [17] using the following broad search string: “(pregn*) AND (nicotin*) AND NOT (rat OR rat* OR mouse OR mice OR goat OR goat*)”. In addition to the search, further articles were snowballed from citations in found articles. We manually narrowed the results to relevant epidemiologic studies.

### Inclusion and exclusion criteria

Based on titles and abstracts, articles on the following topics were excluded:

- Studies that did not include human exposure to a reasonably well-defined smoke-free nicotine product,
- Studies that did not include a pregnancy health outcome variable; this excluded endpoints of smoking cessation, perceptions, attitudes, effects on the women, and cost of interventions (note that as discussed below, the RCTs were really only useful as studies of smoking cessation, but reported birth-outcome statistics so were included),
- Studies that did not appear in an academic journal, could not be found or were not reported in English, Swedish, Danish, or Norwegian.

Following the initial exclusion, investigating full articles, articles were excluded if

- The described methods were inadequate to be confident of what exposure and/or outcome was being reported.

MG and a research associate each reviewed each search result. For four articles, there was initial disagreement about inclusion. All reported associations of pregnancy outcomes and smoke-free nicotine product use are included in the following results.

## Results

**Fig. 1:**
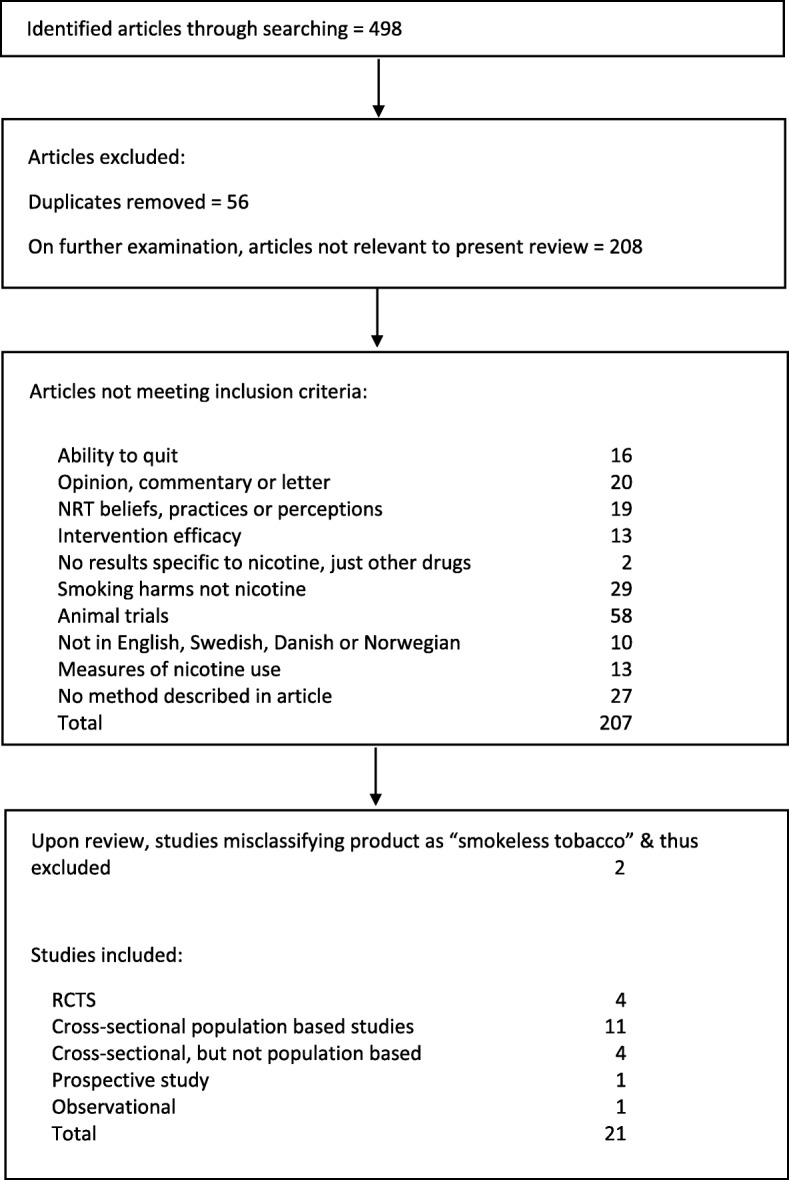
Flow diagram of article selection

Almost 500 studies were found in the initial search (Fig. 1). About half were ignored as they were clearly not relevant to the current review based on their title or they were duplicates. A further 207 did not meet the inclusion criteria. Two further studies were excluded upon review. One reported on Indian products [18], likely containing various substances whose effects are known to be fundamentally different from using proper smokeless tobacco, and the other was conducted in South Africa [19] where a variety of Western, South Asian, and other dip products are widely used, and it was impossible to determine from the paper which constituted the exposures. Twenty-one studies (26 publications) remained to review.

### Descriptions of studies reviewed

The included studies are described in Table 1. Twelve studies looked at NRT use [20–36], 7 at snus use [37–43], and 1 looked at vaping [44]. One study reported on Alaskan iq’mik, a smokeless tobacco mixture that includes a substantial quantity of wood fire ash [45]. While this is not among the products recommended as a low-risk alternative to smoking, it is well-defined and is not necessarily different from normal smokeless tobacco in terms of pregnancy outcomes, so it was included. Four of the studies were randomized trials (RCTs) of NRT products, reported in 8 published articles [20–27], 1 was a prospective cohort study [43], 1 was an observational study [35, 36], and the remainder were retrospective cross-sectional studies [28–34, 37–42, 44, 45].

**Table 1:** Description of studies

Citation	Sample size	Loc^1^	Pop^2^	Design^3^	Data^4^	Condition ^5^	Outcome measure^6^	Adjusted for^7^
Randomized controlled studies								
[20–24]	1010	Eng	White	D_Bl, Pl	SR, CO	Patch, placebo	BW, GA, PT, SB, NICU Ad, ApgS, MF, CD	Recruitment centre
[25]	194	USA	Hispanic, White	D_Bl, Pl	SR, CO, CR, MR	Gum, placebo	BW, GA, ApgS, PT,	Normal distribution only
[26]	181	USA	White	NB	SR, CR	CBT, CBT+NRT	BW, GA	Pre-term birth
[27]	23	USA	White	Cr	US, BP, Pulse	Patch, smoking	Systolic, diastolic BP, middle cerebral, umbilical or uterine arteries, fetal heart rate	N/A
Cross-sectional population-based studies								
[37]	379,214	NC			MR, SR (MWR)	NSNNU, smoking, snus,	Preeclampsia	Maternal age at delivery, early pregnancy, BMI, parity, and YrsEd. Excluded dual users and multiple births
[38]	1,270,161	Sw			MR, SR (MWR)	NSNNU, smoking, Snus, dual-use	PT	Parity, PPBMI, family situation Maternal age, maternal Country of birth, YrsEd. Excluded multiple births
[39]	609,551	NC			MR, SR	NSNNU, smoking, snus	SGA, Nap	SGA: maternal age, height, parity, education. In addition, for apnea: infant gender, GA, SGA, delivery method
[28]	72,761	Dk			MR, TSR	NSNNU, smoking, combined-NRT	BW	Infant sex, parity, fertility problems, maternal age, SES, MS, PPBMI, maternal weight loss during pregnancy, occupational physical strain, physical exercise, coffee, alcohol, nausea, vomiting, vaginal bleeding, eating disorder, hypertension, PSS, maternal height. Excluded multiple births, pre-28 weeks birth,
[40]	610,879	Sw			MR, SR (MWR)	NSNNU, smoking, snus	SB	Maternal age, PPBMI, parity, YrsEd, chronic hypertension, and pre-gestational diabetes. Excluding women with preeclampsia, antenatal bleeding, and SGA.
[29]	90,165	Dk			MR, TSR	NSNNU, smoking, NRT, dual-use	SB	For the NRT groups: smoking, Maternal age, SES. Excluded: Multiple births, pregnancies ending in hydatidiform mole or ectopic pregnancy, or before 20 completed weeks.
[30]	220,630	UK			MR, SR	Never-smoked, RecNRT, smoking	SB	Maternal age, SES, pre-pregnancy BMI, and diabetes. Excluded multi-births
[31]	76,768	Dk			MR, SR	NSNNU, smoking, NRT	MF	Not specified for NRT group
[32]	192,498	UK			MR, SR	NSNNU, smoking, RecNRT	MF	Age, SES, maternal diabetes, asthma, mental illnesses, and multiple births. Excluding: SB
[41]	975,866	Sw			MR, SR	NSNNU, smoking, snus	MF	age, parity, education, living with father-to-be, hypertension, diabetes, PE, sex of newborn, birth (singleton or multiple)
[33]	63,128	Dk			TSR	NRT, NSNNU, smoking	Colic	Maternal age, parity, daily coffee consumption, weekly alcohol consumption, binge-drinking, and education and SES
Cross-sectional non-population-based studies								
[44]	129				MR	Vaping	BW, PT	Not specified
[34]	5716	USA	White		SWQ, BC	RecNRT, never-smoked, smoking	BW, PT	Age, PPBMI, race/ethnicity, education level, MS, income, MA, parity, alcohol, and maternal weight gain during pregnancy. Excluded: no PNC, no information on MS, MA, or alcohol
[42]	23,542	Sw			MR, SR (MWR)	NSNNU, smoking, snus	BW, PT, PE	Maternal age, GA, BMI, parity, infant sex, excluded, multiple births, stillbirths
[45]	41	USA	AN	CS^8^	SI, LipTool	NSNNU (C), Iq’mik,	GA, BW, ApgS, LipTool	Not stated
Prospective study								
[43]	56	Sw		Pilot	SR, COT	Snus, NSNNU, smoking	CVD	BW, baby’s sex, mother’s weight, parity, gestational week at delivery, GA, and BW
Observational pilot study								
[35, 36]	6	USA	White	Pilot	PE, CV	N/A	All	Not specified

^1^Location: *Eng* = England, *Dk* = Denmark, *NC* = Nordic countries, *No* = Norway, *Sw* = Sweden, *USA* = United States of America, *UK* = United Kingdom

^2^Ethnicity of majority population if given: *White* = classified as “white” in the publication, *H* = Hispanic, *AN* = Alaskan native

^3^Additional design features: *Cr* = cross over RCT, *D_Bl* = double-blind, *L* = longitudinal, *LipTool* = Lipsitz tool, *NB* = not blinded, *Pl* = placebo, *Ps* = prospective

^4^*BC* = birth certificate, *BP* = blood pressure, *CO* = carbon monoxide reading, *CR* = cotinine (urine or hair) reading, *MR* = medical records, *MWR* = midwives records, *SI* = scientifically structured interviews, *SR* = self-report (without verification or with non-scientific interviewers), *SWQ* = self-completed written questionnaire, *TSR* = telephone self-reports

^5^Comparison groups: *CBT* = cognitive behavioural therapy, *Combined-NRT* = more than 1 NRT, *ExS* = smokers who quit smoking and did not use any nicotine products while pregnant, *N/A* = not applicable, *NSNNU* = no smoking no nicotine use, *RecNRT* = recommended to use NRT

^6^Outcome measures: *ApgS* = APGAR score, *BW* = birth weight, *CD* = caesarean delivery, *CV* = cardiovascular, *GA* = gestational age, *MF* = malformations, *NAp* = neonatal apnea, *PE* = preeclampsia, *PT* = preterm delivery, *SB* = still birth, *SGA* = small for gestational age

^7^Stating all the variables they adjusted for *MA* = medicaid, *MS* = marital status, *PP* = planned pregnancy, *PPBMI* = pre-pregnancy BMI, *PSS* = partner’s smoking status, *SES* = socioeconomic status, *YrsEd* = years of education

^8^Referred to as a “nonrandomized, clinical observational pilot trial”; however, because the study did not include an intervention, it has been classified as a cross-sectional study

A review of the details of the studies and results makes clear that even this modest count of studies meeting the inclusion criteria is an overstatement of how much information we have.

### Pregnancy outcomes

#### Gestation term

Three RCTs investigated risks of NRT use compared to placebo. The interventions were aimed at smoking abstinence and were analyzed based on intention to treat (i.e., assignment to NRT rather than biological exposure), making the results almost useless for present purposes. Oncken et al. [25] reported a measurable increase in gestation term for pregnant women who smoked who were assigned to nicotine gum compared to a placebo (38.9 weeks vs. 38.0). However, if the association was causal, it would most likely be explained by the slightly greater rates of smoking abstinence and smoking intensity reduction in the treatment group. The effect from that difference is so uncertain that it is impossible to use it to estimate any net effect of the NRT exposure. All that could be said is that any effect of the gum was not sufficient to undo the benefit of less smoking. The SNAP study [21, 22] was a similar trial using nicotine patches and suffered from the same inability to interpret the effects of the biological exposure to NRT, even though smoking abstinence rates were barely greater in the treatment arms. A trivial reduction in preterm birth incidence (8% vs. 9%) was seen in the NRT group.

The third similar RCT (Pollak et al. [26]) had a greater contrast in smoking abstinence, 24% in the group that was assigned ad libitum choice of NRTs vs. 8% for the therapy-only group. Study recruitment was halted by an ethics board because the group that had higher cessation was having worse birth outcomes. The mean gestational age for the treatment group was slightly lower, 37.9 weeks vs. 38.6. A causal interpretation of that association is the seemingly unlikely conclusion that NRT was causing so much reduction in pregnancy term that it more than negated the benefit of smoking abstinence.

An observational study, comparing subjects who had a medical recommendation to use NRT to those who did not, Gaither et al. [34], reported a large increase in the risk of preterm birth for those with the recommendation. The authors, to their credit, implicitly recognized this was almost certainly an artifact of the more dedicated (heavier, less inclined to quit) smokers being more likely to get such a recommendation (known as “confounding by indication”).

Dahlin et al. [38] looked at preterm birth in a large population with substantial snus use and reported substantially increased risk among the snus users vs. those who used no nicotine product. In particular, the adjusted results included “extremely preterm” birth (> 28 weeks), OR = 1.58 (95% CI 1.14–2.21), “very preterm” birth (28–31 weeks), OR = 1.25 (95% CI 0.98–1.59), and “moderately preterm” birth (32–36 weeks), OR = 1.21 (95% CI 1.11–1.31). However, when interpreting the results of this and similar studies, it is important to consider the uncertainty due to non-random error, as discussed below.

A similar study (England et al. [42]) of Swedish birth records reported an increased risk of preterm birth (< 37 weeks) for snus users, adjusted OR = 1.98 (95% CI, 1.46–2.68). The study reported a much more modest association with smoking (OR = 1.57).

The only apparent result for vaping, a conference abstract of a prospective study [44], showed vapers and non-users had similar average gestational ages (39.3 weeks vs. 39.8), and this was also similar for smokers (39.3).

#### Birth weight

The three RCTs also reported birth weight outcomes, and the noted limitations again apply. Oncken et al. [25] reported that the NRT gum group (i.e., those with slightly higher smoking reductions) had a notably higher birth weight, 3287 g (SD = 566) vs. 2950 g (653) for the placebo group. The SNAP study [21, 22] and Pollak et al. [26] reported little difference.

An observational study based on the Danish birth registry and interviews to determine exposure [28] reported NRT overall was associated with approximately no change in birth weight on average compared to non-use. However, the results across different NRT products varied so wildly that the uncertainty of the results is immediately evident, and there is presumably also systematic confounding by unmeasured smoking variables.

The Gaither et al. study [34] reported women who were recommended to use NRT had twice the risk of low birth weight, but the bias from confounding by indication makes this result meaningless. The bias is made clear by observing the elevated risk among smokers without the NRT recommendation was only one-third as large. If the association was naively interpreted as causation, it would mean that NRT use has greater negative effects (by this measure) than its net beneficial effects via causing smoking abstinence.

The aforementioned Swedish birth records study [42] reported a small reduction in mean birth weight for women who used snus, 3529 g (SD 569) vs. 3635 (544) for non-nicotine users. The Gunnerbeck et al. Swedish birth records study [39] reported approximately no difference.

A secondary analysis in a stillbirth study by Wikström et al. [40] reported a small increase in small-for-gestational-age (adjusted OR 1.17; 95% CI (0.98–1.39)), which was much lower than for smoking (ORs of 2.34 (2.21–2.49) and 3.20 (2.94–3.48) for 1–9 and > 9 cigarettes per day).

The conference poster on pregnant women who vaped reported similar results for vaping and non-use, 3482 g (SD 549) vs. 3471 g (SD 504). These both exceeded the average for smokers, 3166 g (SD 502) [44].

#### Stillbirth

The SNAP RCT [21, 22] reported stillbirth statistics but was far too underpowered to be informative (7 total stillbirth outcomes). An extremely large dataset or selection based on outcome (e.g., a case-control study) is necessary for assessing relatively rare dichotomous outcomes.

Wikström et al., a large cross-sectional study in Sweden, focused on this outcome [40]. It reported an increased risk of stillbirth for reported snus use at the first antenatal visit, compared to no nicotine use, with OR = 1.6 (95% CI = 1.1–2.3). This compared to only 1.4 (1.2–1.7) for smoking up to 9 cigarettes/day, and 2.4 (2.0–3.0) for smoking more heavily.

A study focusing on this outcome using the Danish birth cohort [29] reported a protective hazard ratio of 0.67 (95% CI 0.21–2.08) for use of NRT use vs. those who did not use nicotine. Oddly, for those who both smoked and used NRT, the hazard ratio was still in the protective direction (0.83; 95% CI 0.34–2.00). For exclusive smokers, the results were 1.46 (1.17–1.82). A study in the UK [30] looked at prescriptions for NRT and reported an adjusted OR of 1.35 (95% CI 0.91–2.00) compared to non-smokers. The result for smokers was similar, and this is another example of confounding by indication, where the measured exposure variable may better predict smoking intensity than it does switching to NRT.

#### Preeclampsia

Smoking is generally believed to be protective against preeclampsia. It is not clear whether other use of nicotine has similar effects.

The aforementioned Swedish medical records study [42] reported an adjusted odds ratio for snus use, compared to no nicotine use, of 1.58 (95% CI 1.09–2.27). As an apparent secondary analysis in their stillbirth study, Wikström et al. [37] reported the predicted protective effects for smoking, but not for snus use, with OR = 1.11 (95% CI 0.97 to 1.28). Women who started using snus after the first antenatal interview (using it at their 30–32-week visit) had an OR of 0.93 (0.56 to 1.57), which might suggest some protective effect, lesser than that from smoking, is being obscured by confounding.

Wright et al. [35, 36] had 6 pregnant women who had been unable to stop smoking attend a clinic for observation while wearing a NRT patch over 11 and 21 h, respectively. The monitoring did not find any measurable effect of the NRT patch use on umbilical artery Doppler readings or maternal uterine activity.

#### Malformations

Gunnerbeck et al. [41] also looked at oral cleft malformations using Swedish national birth records. They reported elevated rates for women who used snus at their first antenatal visit, adjusted OR 1.48 (95% CI 1.00–2.21) or smoked, adjusted OR 1.19 (1.01–1.41). The greater increase compared to smoking, as well as the protective associations for those who ceased use in the three months before their first visit (adjusted odds ratio 0.71 (0.44–1.14) for snus use and 0.88 (0.73–1.05) for smoking) make it difficult to interpret these results as causal.

Using Danish national mother-child hospital records, Morales-Suárez-Varela et al. [31] looked at 19 different congenital malformations (major and minor) in a large cohort of Danish births. There was a trivial positive association with smoking, with oral cleft malformations standing out as more strongly associated. An analysis of the small number of exclusive NRT users appears to not have been part of the original protocol and was data-driven (i.e., they apparently chose to report this because it was the outlier, as will almost inevitably exist for some combination of variables in a dataset due to random error). They reported an OR of 1.61 (95% CI 1.01–2.58) for all malformations, dropping to 1.13 (0.62–2.07) when limited to major malformations.

Dhalwani et al. [32] looked at NRT prescribing and major congenital malformations in primary care records of a cohort of UK births. As with the other prescribing-based analyses, there is no measure of biological exposure to nicotine and almost certain confounding by indication. They reported an adjusted OR of 1.12 (99% CI 0.84–1.48) for prescribing vs. non-smokers. This was also elevated compared to smokers (adjusted OR 1.07 (0.78–1.47)).

#### APGAR scores

The SNAP [22] and Oncken et al. [25] NRT RCTs reported trivial differences from the null. The above caveats about the RCTs apply. The study of Alaskan women who used iq’mik [45] found a trivial difference compared to either non-nicotine users or smokers.

#### Cardiovascular outcomes

Oncken et al. [27] conducted a crossover study, assigning pregnant women (24–36 weeks gestation) who smoked to use nicotine patches temporarily. They reported similar maternal nicotine levels and similar fetal artery resistance indices for the NRT and smoking compared to baseline. They reported that one secondary comparison (loss of fetal heart rate reactivity) was elevated for the NRT exposure compared to smoking but conceded this was a data-driven observation.

Another secondary analysis of the Wikström et al. stillbirth study [40] reported a reduced risk of gestational hypertension at the first antenatal visit for snus use vs. no nicotine use (adjusted OR 0.89; 95% CI (0.68–1.15)). This compared to 0.66 (0.61–0.71) and 0.51 (0.44–0.58) for women who smoked 1–9 and > 9 cigarettes per day.

A small prospective study [43] looked at two markers of infants’ cardiac autonomic regulation, which the authors suggest predict infant death and represent morbidity in themselves. They reported similar substantial elevations for various comparisons for snus use and smoking during pregnancy as compared to no nicotine use (in the range of double or triple the risk).

The aforementioned small Wright et al. experiment [35, 36] reported no measurable difference in fetal heart rate between when women who smoked were switched to a nicotine patch.

#### Respiratory outcomes

The SNAP [20, 23] RCT reported a small difference in infant respiratory symptoms (OR 1.3; 95% CI 0.97–1.74). The usual caveats about the inability to determine the actual causal contrast (smokers being assigned to use NRT, with complicated effects on actual product usage) apply.

The aforementioned Gunnerbeck et al. Swedish medical birth record study [39] also looked at neonatal apnea and found an approximately doubled risk (after inappropriately trying various models and presumably picking one that suggested a particularly strong association) when women used snus during pregnancy, compared to non-nicotine users, which was higher than the elevated risk associated with smoking.

#### Neurobehavioral effects

The Alaskan iq’mik study [45], which was focused on this outcome, used the Litsitz scale, a collection of measures that might be associated with neonatal nicotine withdrawal (first 72 hours after birth). It reported a substantial increase in that score for iq’mik use, similar to that for smoking, compared to non-nicotine users. However, the clinicians doing the scoring were not blinded to the women’s tobacco use status, creating an obvious potential for biased measurement error, a concern that is further supported by the negative association between the score and cord blood nicotine or cotinine.

#### Colic

Milidou et al. [33] used data from maternal interviews to look at colic prevalence at 6 months by maternal product use during pregnancy. They reported that exclusive NRT use had an adjusted OR of 1.6 (95% CI 1.0–2.5) compared to no nicotine use. This exceeded the adjusted OR of 1.3 (95% CI 1.2–1.4) for exclusive smoking and was similar to that for using both products (1.6; 95% CI (1.3–1.9).

#### Uncertainty of the estimates

The paucity and (inevitable) inconsistency of the available data is sufficient to see that we cannot quantify the pregnancy outcomes risk from smoke-free nicotine and tobacco product use. The available results—as well as any further similar results that will appear in the foreseeable future—are inherently too imprecise to provide a quantitative estimate. Even results that are typically naively presented as supporting the same conclusion flatly contradict one another. For example, for the two Swedish medical records studies of preterm birth, one of them estimated snus doubles the risk while the other puts the increase at around a third. This pattern is often naively described as them agreeing that there is an increased risk, but in fact, the second estimate more clearly rejects the accuracy of the first estimate than it does the conclusion that there is no increase. At least one of these estimates is wrong.

The only reason we have reasonably useful estimates of birth effects from smoking is that the question is easier and has been veritably besieged with studies determining the effects of smoking while pregnant. Significantly, the relative homogeneity of the exposure makes all these measures of roughly the same exposure. That is, most smoking is and has been about the same exposure, except for intensity (which remains an under-measured variable that adds a lot of unacknowledged uncertainty). But the smoke-free product exposures differ importantly not only in intensity but by product, as well as over time.

The observational studies suffer from all the usual problems of such studies. These include uncontrolled or under-controlled confounding, particularly including the easily observed confounding by indication in some of these studies. The usual problems of simple measurement error (e.g., someone just typing the wrong data) are compounded by data being collected for a different purpose and thus perhaps not measured in the ideal way for the study. Selection bias is less of a problem than usual for the population-wide studies that appear here, but still might exist (e.g., not everyone attends their assigned medical visits, even in Scandinavia).

To illustrate the magnitude of this uncertainty that is commonly ignored, consider one result from the England et al. [42] study, chosen arbitrarily from among the estimates from those retrospective medical records studies that did not suffer from the obvious confounding by indication. The paper reported an increased risk of preterm birth for snus users, with an adjusted OR = 1.98 (95% CI, 1.46–2.68), and a smaller association for smoking (OR = 1.57). The reasonably narrow confidence interval reflects only random sampling error, not other sources of uncertainty (though it alone represents almost as much uncertainty as we started with). The “adjusted” is often interpreted by naïve readers as saying that there is little or no residual confounding. But the data is limited to medical records and lacks information on other relevant behaviors and non-medical conditions. Most potential confounding is simply ignored. That is, it is not adjusted for.

To emphasize how much uncertainty this adds, consider a calculation based on Lash et al.’s textbook [46]. (One of us (CVP) developed more involved methods [47–49] which Lash has also expanded upon, but this is sufficient to illustrate how much unreported uncertainty there is and is easily replicated using the author’s spreadsheet at [50]). We know that people who used tobacco products at this time (1999–2000), particularly including women who continued to use the products during pregnancy, are systematically different from those who did not in ways that are not measured in the medical records. Consider a single hypothetical latent dichotomous socioeconomic (SES) variable, “low SES,” that is associated with the choice to use snus and with the risk of premature birth (e.g., via having greater stress, other unhealthy behaviors, or physical demands). If low SES has an OR of 1.5 for preterm birth and it is associated with snus use, with 2/3 of snus users being positive for low SES vs. only 1/10 of those who use no nicotine, then the adjusted OR drops to 1.55, about half the increased risk originally estimated. The adjustments from the original study are ignored because it turns out they changed the effect estimate by less than 1%. This is not to suggest that these are the best choice of inputs for this correction, but merely to illustrate how much the point estimate moves based on plausible uncontrolled confounding. Perhaps this explains the fairly implausible higher risk compared to smoking. But, other determinants exist, for instance, smoking in Sweden is strongly associated with non-Nordic ethnic status.

The addition of other latent confounding variables creates additional uncertainty, as do the possibilities of measurement error and selection bias. These accidental biases do not even consider the possibility of the common practice on the part of epidemiology researchers to make choices about their statistical model to generate a more “interesting” result (sometimes called “researcher degrees of freedom” or “publication bias in situ”). The researchers chose one of the many exposure status definitions available to them, picked a cutoff value for preterm, and made many choices about exclusion criteria. There is no indication that these were pre-specified, and thus any of them might have been chosen because they moved the effect estimate in a particular direction compared to some alternative modeling choice that would seem equally reasonable. In short, the quantitative estimates we have are not nearly as good as they look when interpreted naively.

The RCTs are even less informative than the observational studies. For present purposes, we are interested in the effect of using NRT. But what these give us instead is an estimate of effect of smokers being assigned to use NRT, filtered through the various causal pathways of the effect of that on smoking cessation, the effect of smoking intensity on compliance and quitting without using the NRT, plus the actual effect of using NRT. When analyzed based on intention-to-treat, as is the usual preference, RCTs could estimate only the direction of the effect on a birth outcome compared to smoking—i.e., whether the effect of the substitute is measurably less than smoking or not. But even this depends on the intervention having a substantial effect on causing enough substitution for smoking to get a biological contrast, which was not the case for the existing literature. Any quantitative estimate (not merely the qualitative “it seems to be less than for smoking”) would require an impossibly accurate estimate of the effect of smoking and how it differs among those who quit or switched vs. those who continued smoking. The RCT data could be analyzed as an observational study of consumption choices, which could provide a better estimate of the real effect of the NRT, but this is rarely done, and it would still be a very small observational study with one more source of potential confounding than naturally occurs (the effect of whether someone is inclined to comply with their assignment). Whilst we included the RCTs in this review, for completeness, they do not provide useful measures of the effects of NRT use on birth outcomes.

## Conclusions

This review reaffirms the central conclusions of the previous reviews which were that there was insufficient evidence to quantify any risks. Despite more research having been done on these topics, the state of knowledge remains the same. The nearness of all the results to the expected range does provide some assurance that nothing wildly unpredicted is occurring. The epidemiology does not give us reason to reject our prior belief that the effects of smoke-free nicotine products on pregnancy outcomes are less than those from smoking, but more than nil. This is valuable because it strengthens our confidence in what we already believed.

There are some outlier results that fall outside those bounds in each direction, but the inevitable uncertainty and sometimes obvious bias in the studies should leave a lot more weight on our prior knowledge than on those results. It would require a *lot* of evidence to convincingly argue that NRT and snus have larger detrimental effects than smoking. Smoking includes all the exposures from NRT and snus, and most of those from e-cigarettes, in addition to a large dose of carbon monoxide, particulate matter that passes into the bloodstream, and much higher levels of various other toxicants.

If we were to naively interpret the estimates from some of these studies, they would suggest that pregnant women who smoke should not be prescribed NRT because that causes harm, and that if they use snus they should switch to smoking. It is difficult to imagine either of those is good advice. It is more reasonable to doubt a causal interpretation of the study results.

Another conclusion we can draw is that the usual generic recommendation for “more research” would be misleading. If the existing literature included four more studies using the same methodology for each study in this review, we would still have a very tenuous set of estimates. A realistic quantity of “more research” along the lines of the existing research is simply not going to tell us much. A corollary of this is that when the next such study is published, and the press release claims “now we know X,” that is simply false.

There is simply too much uncertainty in each individual estimate, even apart from the studies that are so uninformative that they should just be ignored. Coupled with the multiple endpoints of interest and myriad exposures (note that this does not just mean the list of product categories, though that is long enough, but also specifics of the products, dosage, and other variables), a literature an order of magnitude larger than what exists, and with average quality of at least that of the 75th percentile of quality for this literature, would only begin to be informative.

Typically, we think of toxicology and similar scientific methods as providing a reality check that nothing wildly beyond our expectations is happening. We then depend on epidemiology to provide quantification. But in the present case, the available and foreseeable epidemiology offers little more than the reality check. For the foreseeable future, our best assessment of the effects of smoke-free nicotine and tobacco product use on pregnancy outcomes will have to be informed by the rough gestalt of various sciences and extrapolation from known (minimal) effects on the consumer herself.

The lack of useful information from the randomized trials is particularly telling. Naïve readers of health science often suggest that RCT results are the most informative (full stop—i.e., about anything for which the study collected results). Realistic trials in this space will provide barely any useful information about the effect of the biological exposure. Subjects can only be assigned to a treatment, not to a biological exposure, and it is likely that the biological exposure will vary so little between treatment groups (as observed in the existing literature) to preclude useful conclusions about its effects. It might be the case that RCTs involving a vaping treatment arm would be more informative, since some interventions that encouraged vaping have shown a very high rate of complete switching. But even that, analyzed as intention to treat, would leave us guessing about how to correct for the effects of residual confounding from smoking. “More RCTs are needed” is the worst possible advice that could be offered in this context. At the same time, even the observational studies based on the rare big datasets suffer from the problems demonstrated in the uncertainty analysis.

Pregnant women and those who advise them would like to be able to assess the risks of using smoke-free nicotine and tobacco products, not just have vague notions of what is better and worse. “There is probably a cost” is not terribly useful for decisions that must be weighed against other costs and benefits. But that is where we are. The use of smoke-free nicotine products almost certainly has less effect than smoking on pregnancy outcomes (most of which are negative, but there are some positive effects), but any use of nicotine is probably worse for the fetus than none. This review reinforces both the validity of that advice and the fact that more precise advice cannot be offered. There is certainly no basis for offering the advice that the benefits of avoiding these products exceed the costs without understanding the costs for any particular individual. This review demonstrates that the evidence does not support denying pregnant women the use of smoke-free products if the alternative is that she would continue to smoke.

## Data Availability

Data sharing is not applicable to this article as no new datasets were generated or analyzed during the current study.

## Abbreviations

AN: Alaskan native
ApgS: APGAR score
BC: Birth certificate
BP: Blood pressure
BW: Birth weight
CBT: Cognitive behavioral therapy
CD: Caesarean delivery
CI: Confidence interval
CO: Carbon monoxide reading
Cr: Cross over RCT
CR: Cotinine (urine or hair) reading
CV: Cardiovascular
D_Bl: Double-blind
Dk: Denmark
Eng: England
ExS: Ex-smoker
GA: Gestational age
H: Hispanic
L: Longitudinal
LipTool: Lipsitz tool
MA: Medicaid
MF: Malformations
MR: Medical records
MS: Marital status
MWR: Midwives records
N/A: Not applicable
Nap: Neonatal apnea
NB: Not blinded
NC: Nordic countries
No: Norway
NRT: Nicotine replacement therapy
NSNNU: No smoking no nicotine use
OR: Odds ratio
PE: Preeclampsia
Pl: Placebo
PP: Planned pregnancy
PPBMI: Pre-pregnancy body mass index
Ps: Prospective
PSS: Partner’s smoking status
PT: Preterm delivery
RCT: Randomized controlled trial
RecNRT: Recommended to use NRT
SB: Still birth
SD: Standard deviation
SES: Socioeconomic status
SGA: Small for gestational age
SI: Scientifically structured interviews
SR: Self-report
Sw: Sweden
SWQ: Self-completed written questionnaire
TSR: Telephone self-reports
YrsEd: Years of education
